# Health-Related Quality of Life Based on EQ-5D Utility Score in Patients With Tuberculosis: A Systematic Review

**DOI:** 10.3389/fphar.2021.659675

**Published:** 2021-04-14

**Authors:** Hae-Young Park, Hyo-Bin Cheon, Sun Ha Choi, Jin-Won Kwon

**Affiliations:** ^1^ BK21 FOUR Community-Based Intelligent Novel Drug Discovery Education Unit, College of Pharmacy and Research Institute of Pharmaceutical Sciences, Kyungpook National University, Daegu, South Korea; ^2^ Lung Cancer Center, Kyungpook National University Chilgok Hospital, Daegu, South Korea

**Keywords:** tuberculosis, quality of life, EQ-5D, systematic review, meta–analysis

## Abstract

**Background:** Tuberculosis (TB) has significant effects on patients’ health-related quality of life (HRQOL) and this study was conducted to evaluate the HRQOL based on EQ-5D utility score according to various health statuses of TB patients.

**Methods:** A systematic literature review was conducted to select articles on HRQOLs outcomes of TB patients since 2000. A total of 1,710 articles were searched for primary screening and seven studies that directly assessed all types of TB patients using the EQ-5D were finally selected.

**Results:** The EQ-5D scores of TB patients were in the ranges of 0.43–0.70. After the completion of TB treatment, the utility weights increased to the ranges of 0.88–0.98 and the EQ VAS values showed similar trend as the results of the EQ-5D. The EQ-5D score for multi-drug-resistant TB was very low at 0.51 during treatment but increased to 0.88 after the treatment was completed. The utility weights of latent TB were not significantly different from those of the general population.

**Conclusion:** This study showed that the HRQOL based on the EQ-5D utility score of TB patients has significantly decreased, and the TB treatment has a significantly positive effect on the quality of life of patients.

## Introduction

As of 2018, South Korea had 66 new cases [95% confidence interval (CI): 61–71] of *tuberculosis* (TB), and 4.8 deaths (95% CI: 4.5–5.0) due to TB per 100,000 populations, ranking first and second, respectively, among countries of the Organization for Economic Co-operation and Development, making TB control a top priority for national disease control and prevention (KCDC, 2019; WHO, 2019). Therefore, South Korea has been implementing a mid-to-long-term TB management plan in line with the “End TB Strategy” by the World Health Organization to terminate TB by 2030 (WHO, 2016; KCDC, 2018; MOHW, 2018). The major indicators of the plan, such as the incidence of TB and the success rate of its treatment are showing a favourable trend; however, they still have not met the target level, and the Korean government is reinforcing every policy to further improve them.

TB is caused primarily by an infection of the *Mycobacterium tuberculosis* complex. *M. tuberculosis* often remains latent for a considerable period of time and multiplies slower than other bacteria. For this reason, TB patients need more than 6 months of continuous and long-term treatment compared to patients with other bacterial infections (KCDC, 2017). Failure to properly comply with the long-term treatment guidelines may result in TB recurrence or an incidence of multidrug-resistant *tuberculosis* (MDR-TB), and in some cases, extensively drug-resistant *tuberculosis* (XDR-TB). MDR-TB requires more than 2 years of treatment when it occurs, and the number of administered drugs and administration methods are more complex as they are accompanied by hospitalization or surgery. This long-term treatment of TB causes physical problems and extreme psychological stress that lowers the health-related quality of life (HRQOL) of patients, which can also reduce the outcome of TB treatment (Datta et al., 2020).

Therefore, every effort is being made to develop new regimens and drugs that increase the treatment success rate and shorten the treatment period and several new health technologies are being introduced in the market (Schito et al., 2015). However, new health technologies, such as the strengthening of direct-observation or the administration of new drugs effective in drug-resistant *M. tuberculosis*, usually accompany an increase in cost, which need to be evaluated compared to increased HRQOL utility in patients (Bloom et al., 2017). HRQOL in medical outcome studies with *tuberculosis* patients has most commonly been evaluated using the 36-item short-form health care survey (SF-36) (Bauer et al., 2013; Brown et al., 2015). The SF-36 evaluates the patient’s health status across eight dimensions, making it suitable for an in-depth assessment of the patient’s HRQOL, but utility scores cannot be directly calculated from the SF-36 without converting to the SF-6D or mapping onto the EQ-5D because the score algorithm is not based on individual preferences (Brazier et al., 2002). On the other hand, the EQ-5D is the most commonly used HRQOL assessment tool in economical appraisals (Kennedy-Martin et al., 2020) because the EQ-5D can quantifying utility values based on societal preferences, and is also the most validated instrument in many countries (Qian et al., 2020).

Korea has the highest burden of TB among the Organization for Economic Co-operation and Development countries and has introduced various policies and new drugs with the goal of ending TB by 2035 (Cho, 2018; Min et al., 2020). In Korea, cost-effectiveness evaluation is mandatory to list new drugs under reimbursement scheme (Byun et al., 2016; Park et al., 2016), and economic evaluations to set up policies are increasing, but the EQ-5D data of Koreans has yet to be published. Thus, this study was conducted to evaluate the HRQOL based on the EQ-5D utility score, according to the health statuses of TB patients, through a systematic literature review. The results of this study are expected to be used for the economic evaluation of new health technologies for TB in Korea as well as other countries in the future.

## Methods

### Search Strategy

The process of literature search and screening was carried out in accordance with the guidelines of Preferred Reporting Items for Systematic Reviews and Meta-Analysis (PRISMA) (Moher et al., 2009). The target patients were TB patients including MDR-TB. Intervention and comparator were not limited specifically, and the outcomes were set HRQOL. Although the target outcome of this study is EQ-5D, we broadly set the search terms to include outcomes involving HRQOL-related terms and chose the EQ-5D studies during the screening and selection process. PubMed, Cochrane Library, and EMBASE were the main databases searched, while KoreaMed and RISS were also used as databases for local literature searching. The time scope was set from January 1, 2000 to January 10, 2020. Key search terms used were expanded terms associated with TB, HRQOL, preference, EQ-5D, and instruments. More specifically, the terms for EQ-5D outcome were set to EQ-5D, EQ5D, and EuroQol for PubMed searching, according to the EuroQoL Group’s guidance^1^ (For the details of search terms and results, refer the Supplementary Table S1).

### Literature Selection

The literatures searched were independently reviewed by two researchers according to the following selection criteria: 1) The indication of the study population should be TB including MDR-TB, 2) HRQOL should be evaluated by the EQ-5D, 3) The study should be original research and exclude if it is a review, case-report, or abstract 4) The article should be written in English or Korean. A two-stage screening process was applied, searched literatures were firstly screened by title and abstract reviews, and then a full-text screening was conducted in the second stage. In the event of disagreement between the two researchers, the final literature was selected through mutual discussion. EndNote, Version X9, Clarivate Analytics, 2018 were used in the literature selection process.

### Data Extraction and Quality Assessment

Data were extracted in standardized form from the finally selected literatures for the following items; Socio-demographic data, type of TB, study design, inclusion/exclusion criteria, EQ-5D instrument version, value set used to calculate EQ-5D index score, as well as the overall and subgroups’ study outcomes. The quality of the studies was evaluated independently by the two researchers using both the Risk of Bias Assessment Tool for Non-Randomized Studies (RoBANS) (NECA, 2011) and the Newcastle-Ottawa Quality Assessment Scale (NOS) (Wells et al., 2020).

### Data Collection and Analysis

The results of the selected studies were analyzed through a meta-analysis or qualitative data review. Quantitative synthesis was conducted on the four studies, which had utility scores for individual patients both before and after TB treatment. The EQ-5D questionnaire comprises of the EQ-5D profile data across five dimensions and the EQ VAS. The questionnaires on the profile data currently have three versions; EQ-5D-3L (three levels of problems in each dimension), EQ-5D-5L (five levels of problem), and EQ-5D-Y (developed for children and adolescents). The EQ-5D profile data are converted to a single number of EQ-5D value using a value set, a scoring system, which is usually country specific and reflects differences in societal preferences. The EQ VAS rates patients’ own assessment on their overall health using Visual Analogue Scale from 0 (worst health imaginable) to 100 (best health imaginable). Therefore, EQ VAS score provides complementary information to the EQ-5D profile without being affected by the value set or EQ-5D instrument version (Devlin et al., 2020). Since the value set and EQ-5D instrument version can affect the EQ-5D index score, the meta-analysis of the EQ-5D score was conducted on the two studies which had same value set and EQ-5D instrument version, while meta-analysis of the EQ VAS included all four studies. In the meta-analysis, the mean difference (MD) before and after *tuberculosis* treatment by study was calculated and the average MD estimates for total studies and its 95% CI were presented. The Higgin’s I^2^ statistics were higher than 75% and thus, a random effect model was used. The statistical significance of the summary estimates was determined on the basis of a *p*-value of 0.05. Data integration and synthesis in the meta-analysis was conducted using *Review Manager (RevMan) Version 5.4 and The Cochrane Collaboration, 2020*. The utility weights for latent TB and MDR-TB were not able to synthesize quantitatively, so only qualitative review results were presented.

## Results

### Literature Search and Selection

**FIGURE 1:**
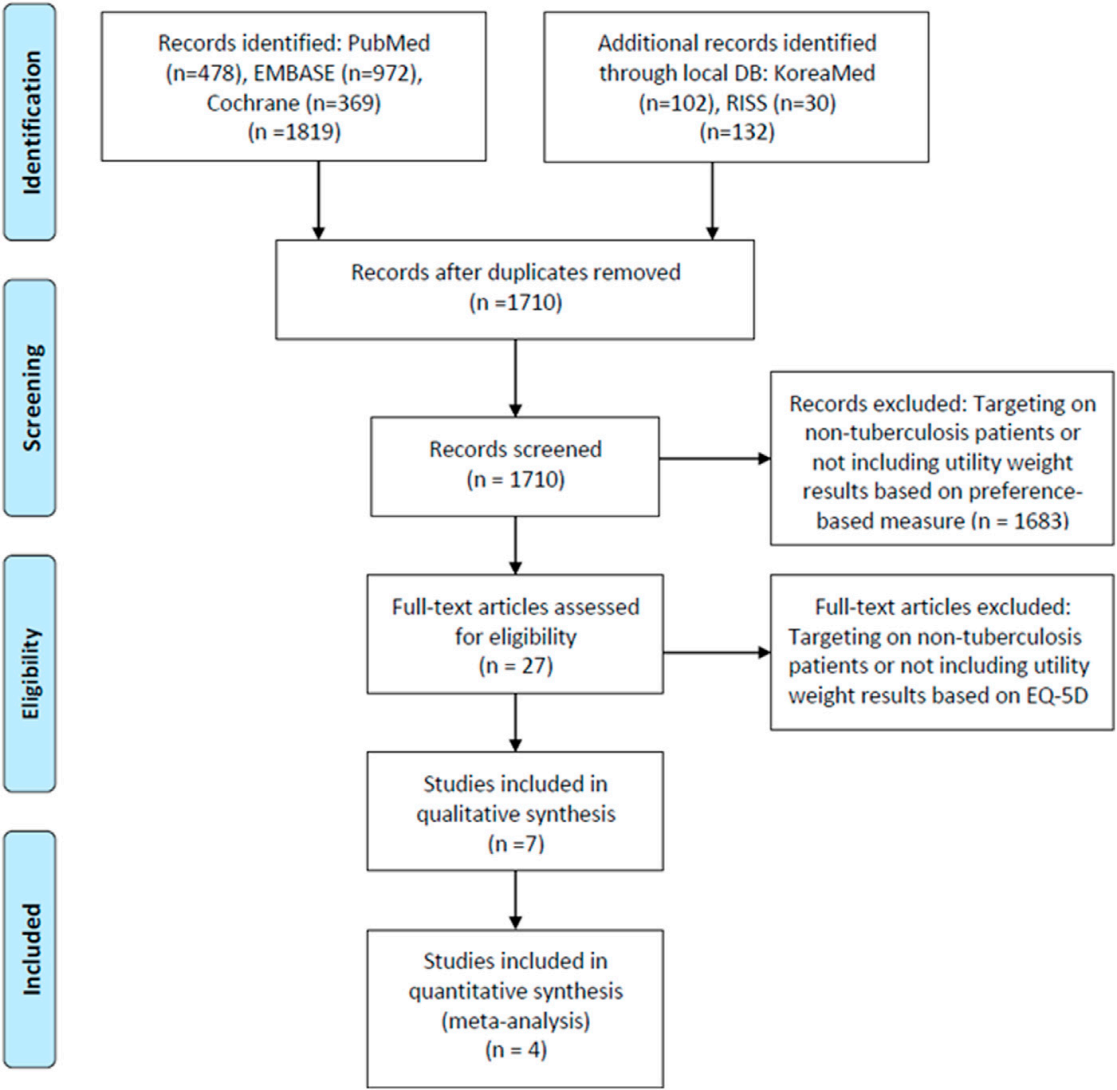
Flow of study selection.

A total of 1819 articles were searched through PubMed, Cochrane Library, and embase, while 132 were searched through domestic databases. After removing a total of 241 duplicate articles, 1710 were included in the primary literature screen process. In the first screen stage, a total of 1,683 articles were excluded as they did not include specific HRQOL results using EQ-5D instruments. A full-text screening was conducted for 27 articles, and finally, seven articles (Dion et al., 2004; Awaisu et al., 2012; Kittikraisak et al., 2012; Kastien-Hilka et al., 2017; Saleem et al., 2018; Zarova et al., 2018; Shedrawy et al., 2019) were selected which met the inclusion/exclusion criteria (Figure 1).

### Features of Finally Selected Studies

Feature of included studies were presented in Table 1. All studies but Dion 2004 have been conducted since 2006, with five studies in Asia and Africa and two in Canada and Sweden. In the Shedrawy 2019 study, only latent TB patients were targeted, while the rest of the studies targeted TB patients, and Kitikraisak 2012 was the only study to include MDR-TB patients. Zaroba 2018 and Shedrawy 2019 were cross-sectional studies and the others were cohort studies. Health-related utility weights were assessed using only the EQ-5D in the Dion 2004 study, all of the other studies were measured using both the EQ-5D and EQ VAS. Kitikraisak 2012 and Shedrawy 2019 used the Thai and Swedish value sets, and Kastien-Hilka et al. (2017) used United Kingdom and Zimbabwe sets, while others used United Kingdom values set to calculate EQ-5D index score; EQ-5D version was EQ-5D-3L in most studies except in the study of Kastien-Hilka et al. (2017), which used EQ-5D-5L. The most common average age range of patients was between 30 and 40 years, and the overall proportion of men was higher than women except in the Shedrawy 2019 study, in which the ratio of women was higher at 76 percent. The level of education differed from study to study, but the percentage of subjects who received a college education was very low and the percentage of patients with jobs was 27–36% in the four reported studies.

**TABLE 1:** Characteristics of Selected Studies and Subjects.

Study	Country	Study year	Study design	Patient number	Included TB status	EQ-5D version	Value set (tariff)	Ethnicity	Mean or Median Age (SD or IQR)	Male % to N	Education status	Employment
Dion et al. (2004)	Canada	1999	Prospective cohort study	50	TB, LTBI	EQ-5D-3L	United Kingdom	African 34%, Asian 26%, Western 40%	Median 33.8 (26.8–41.3)	64%	Median years of education: 16.0 (IQR: 14.0–18.0)	NR
Awaisu et al. (2012)	Malaysia	2006	Prospective cohort study	46 (control group only)	TB	EQ-5D-3L	United Kingdom	Malay 63%, Chinese 24%, others 13%	Mean 43.1 (14.4)	98%	NR	NR
Kittikraisak et al. (2012)	Thailand	2009	Prospective cohort study	222	TB, MDR-TB and/or HIV	EQ-5D-3L	Thai	Thai (100%)	Median 40.0 (35.0–47.0)	62%	Never attended school 8%, primary school 46%, high school or above 46%	Employed 36%
Kastien-Hilka et al. (2017)	South Africa	2014	Prospective cohort study	131	TB	EQ-5D-5L	United Kingdom, Zimbabwe	Black 90%, others 10%	Mean 35.8, Median 31.0	63%	Primary school 24%, high school or above 76%	Employed 27%
Saleem et al. (2018)	Pakistan	2015	Prospective cohort study	226	TB	EQ-5D-3L	United Kingdom	Pakistani 100%	<36 62%, ≥36 38%	51%	NR	Employed 35%
Zarova et al. (2018)	Zimbabwe	NR	Cross-sectional	332	TB	EQ-5D-3L	NR	NR	Mean 40.1 (12.5)	53%	Primary school 18%, Secondary or above 82%	Employed 32%
Shedrawy et al. (2019)	Sweden	2017	Cross-sectional	108	LTBI	EQ-5D-3L	Swedish	African 61%, Asian 31%, European/others 8%	Mean 29.6 (2.4)	24%	NR	NR

HIV, human immunodeficiency virus; IQR, interquartile range; LTBI, latent tuberculosis infection; MDR-TB, multi-drug-resistant tuberculosis; NR, not reported; SD, standard deviation; TB, tuberculosis

### Quality Assessment

The risk of bias in the selected articles was generally low using the RoBANS and NOS (Table 2). In the RoBANS assessment, the risks of bias for the selection of participant and confounding variables were evaluated as low and uncertain, respectively. The risk for intervention measurement was also low as it was done based on a structured interview. In all studies, the risk for blinding of outcome assessment was evaluated to be high due to the absence of blindness process, and the risk of incomplete outcome data and selective outcome reporting was assessed to be low. NOS appraisal was done for five cohort studies, excluding Zaroba 2018 and Shedrawy 2019. Like RoBANS, the overall risk of bias was low, with a total of six stars of NOS score in all studies. Selection of the non-exposed cohort and assessment of outcome could not receive stars because all studies did not set a non-exposed cohort and the outcome was assessed based on patient self-answering even when the studies used a structured questionnaire.

**TABLE 2:** Risk of bias assessment for selected studies.

		Dion et al. (2004)	Awaisu et al. (2012)	Kittikraisak et al. (2012)	Kastien-Hilka et al. (2017)	Saleem et al. (2018)	Zarova et al. (2018)	Shedrawy et al. (2019)
Risk of bias assessment tool for non-randomized study (RoBANS)								
Selection of participants		Low	Low	Low	Low	Low	Low	Low
Confounding variables		Unclear	Unclear	Unclear	Unclear	Unclear	Unclear	Unclear
Intervention measurement		Low	Low	Low	Low	Low	Low	Low
Blinding of outcome assessment		High	High	High	High	High	High	High
Incomplete outcome data		Low	Low	Low	Low	Low	Low	Low
Selective outcome reporting		Low	Low	Low	Low	Low	Low	Low
Newcastle-ottawa quality assessment scale (NOS)								
Selection	Representativeness of the exposed cohort	*	*	*	*	*	NA	NA
	Selection of the non-exposed cohort	-	-	-	-	-	NA	NA
	Ascertainment of exposure	*	*	*	*	*	NA	NA
	Outcome of interest was not present at start of study	*	*	*	*	*	NA	NA
Comparability	Comparability of cohorts on the basis of the design	*	*	*	*	*	NA	NA
Exposure	Assessment of outcome	-	-	-	-	-	NA	NA
	Enough follow-up for outcomes to occur	*	*	*	*	*	NA	NA
	Non-response rate	*	*	*	*	*	NA	NA
NOS Score	Total stars (max 9)	6	6	6	6	6	NA	NA

### Utility Weights of TB Patients

**FIGURE 2:**
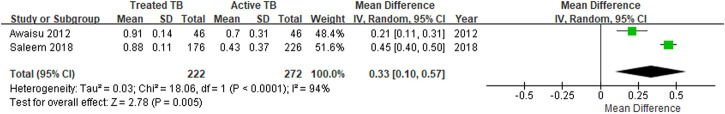
The meta-analysis for the EQ-5D mean differences before and after treatment of *tuberculosis*.

**FIGURE 3:**
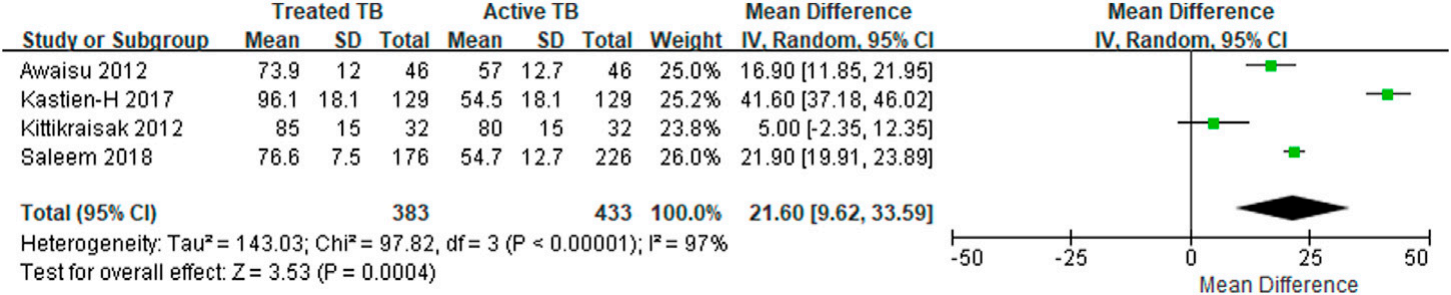
The meta-analysis for the EQ VAS mean differences before and after treatment of *tuberculosis*.

Four studies reported the EQ-5D or EQ VAS scores both before and after TB treatment for the same target patients, and the heterogeneity among the studies was assessed to be high. The MD in the EQ-5D score before and after patients’ treatment for TB increased by an average of 0.19–0.47 compared to patients with an active TB status (Table 3), and the MD from the meta-analysis was 0.33 (95% CI: 0.10–0.57) in the two studies, which used both the United Kingdom value set and EQ-5D-3L (Figure 2). The summarized MD using the EQ VAS was +21.6 (95% CI: 9.6–33.6) (Figure 3). A qualitative summary of the studies not included in the meta-analysis is presented in Table 4. The EQ-5D in the patients with latent TB was very close to the perfect health condition of 1, showing HRQOL conditions that were almost similar to that of the general people. The EQ-5D for the patients with previous TB was 0.83 in the Dion 2004 study alone, higher than active TB and lower than latent TB. The EQ-5D value for MDR-TB was reported only in the Kitikraisak 2012 study, and was very low at 0.51, indicating that MDR-TB significantly degraded the quality of life, but increased to 0.88 after completion of treatment, as with drug-susceptible TB. Similar to the EQ-5D results, the EQ VAS was higher for latent TB patients compared to those for active TB patients, and those with MDR-TB showed a lower value and tended to increase after treatment.

**TABLE 3:** Mean differences of EQ-5D before and after *tuberculosis* treatment.

Study	EQ-5D version	Value set	Active TB			Treated TB			Mean difference	
			Mean	SD	Patient number	Mean	SD	Patient number	Average	95% CI
Awaisu et al. (2012)	EQ-5D-3L	United Kingdom	0.70	0.31	46	0.91	0.14	46	0.21	0.11–0.31
Kittikraisak et al. (2012)	EQ-5D-3L	Thai	0.69	0.22	32	0.88	0.17	32	0.19	0.09–0.29
Kastien-Hilka et al. (2017)	EQ-5D-5L	United Kingdom	0.505	0.328	129	0.980	0.328^a^	129	0.47	0.35–0.60
		Zimbabwe	0.620	0.203	129	0.893	0.203^a^	129	0.27	0.22–0.32
Saleem et al. (2018)	EQ-5D-3L	United Kingdom	0.43	0.37	226	0.88	0.11	176	0.45	0.40–0.50

SD, standard deviation; TB, *tuberculosis*.

^a^ Assumed the same value as the SD of active TB.

**TABLE 4:** Health-related quality of life for tuberculosis patients in various disease stages.

	EQ-5D score (median (IQR) or mean ± SD)			EQ VAS (median (IQR) or mean ± SD)		
	Latent TB	MDR-TB	Other TB status	Latent TB	MDR-TB	Other TB status
Dion et al. (2004)	0.85 (0.80,1.00)	NR	Current TB: 0.80 (0.69, 1.00), previous TB: 0.83 (0.76, 1.00)	NR	NR	NR
Kittikraisak et al. (2012)	NR	On tx: 0.51 (0.39, 0.73), completed tx: 0.88 (0.67, 1.00)	TB with HIV: 0.73 (0.63, 1.00)	NR	On tx: 60 (40, 80), completed tx: 85 (80, 100)	TB with HIV: 80 (70, 90)
Zarova et al. (2018)	NR	NR	Current TB: 0.67 ± 0.24	NR	NR	Current TB: 51 ± 18
Shedrawy et al. (2019)	1.00 (0.79,1.00)	NR	NR	90 (75,100)	NR	NR

IQR, interquartile range; MDR-TB, multi-drug-resistant *tuberculosis*; NR, not reported; TB, *tuberculosis*; Tx, treatment.

### Ethics Committees

This study was based on published data and patients were not directly involved in the entire research process, therefore the need for ethical committee approval and informed consent was waived.

## Discussion

The results of this review show the HRQOL of TB patients based on EQ-5D utility score is significantly lower than that of the general population, and a significant increase in EQ-5D score was shown after completion of TB treatment compared to before the start of treatment. The MDR-TB shows lower EQ-5D value than simple TB, confirming that the progress of treatment and severity of TB affect the HRQOL of TB patients.

In this review, seven studies researched the EQ-5D utility score of active TB patients with the range of 0.43 by the Saleem et al. (2018) study and 0.70 by the Awaisu et al. (2012) study, showing differences in the absolute values between studies. Furthermore, the results of the EQ-5D index scores vary depending on the value set (“tariff”) because each population may show different preferences for various health conditions (Janssen et al., 2019). However, most studies in this review used the United Kingdom value set, and the EQ-5D score of patients who completed TB treatment was not significantly different across all studies; these ranged from 0.88 to 0.98, close to 1.00 (completely healthy), indicating that the treatment of TB significantly affected the HRQOL of the patients. Moreover, since the minimal important differences for EQ-5D-3Ls is estimated to be between 0.03 and 0.08 (Walters and Brazier, 2005; Shams and Pullenayegum, 2019) and the MD in this study is in the range of 0.19–0.47 (Table 3), the results of this study could be considered robust. MDR-TB reported a lower value than drug-susceptible TB at 0.51, and also showed an increase of 0.37 after completion of treatment, indicating that successful treatment of MDR-TB also had a significant effect on improving the HRQOL of patients. The need for clinical and economical appraisal on new health technologies and polices is increasing in countries with heavy TB burden. Unfortunately, studies on the EQ-5D utility score for TB patients are still limited, and thus, the results of this study on utility scores across various health statuses could be a reference for those evaluations.

Treatment of TB requires relatively long-term and complicated methods of drug administration, and adherence and good compliance to treatment have a great impact on the success of patients' treatment and the prevention of recurrence and adverse events. Alipanah et al. reported that increased adherence to TB treatment directly affects the improvement of treatment outcomes through an extensive systematic review (Alipanah et al., 2018). In their meta-analysis to compare the cohort of directly observed therapy (DOT) and self-administered therapy (SAT), the treatment outcomes in the SAT cohort was poorer compared to the DOT cohort with a risk ratio (RR) of 0.81 (95% CI 0.73–0.89) for treatment success, a RR 0.84 (95% CI 0.75–0.93) for adherence, and a RR 4.19 (95% CI 2.34–7.49) for drug resistance. The results according to DOT providers indicated that the DOT provided by health professionals showed better treatment results compared to the DOT by family members (RR 1.79 (95% CI 1.08–2.98) for treatment failure (Alipanah et al., 2018). Based on these results, it is assumed that the compliance and adherence to TB treatment will be closely related to increasing the patient's treatment success rate, changing health conditions, and improving the HRQOL of patients. However, about 50% of patients did not properly comply with the drug administration guidelines based on a survey of TB patients in South Korea (Jung and Hwang, 2018). Therefore, the implementation of good compliance for TB treatment should be better reinforced in consideration of not only improving the patient's treatment success rate but also affecting their quality of life.

In this review, the HRQOL for latent TB was reported in two studies, which showed no significant difference from the general population. The WHO's global estimate of latent TB for the world population is 23% (20.4–26.4%), and the estimate for South Korea is even higher and estimated to be 33% of the total population (Cho, 2018; MOHW, 2018). If they are transferred to TB patients, the disability-adjusted life years due to TB will be increased tremendously. Therefore, in addition to the treatment policy for TB patients, the preventive strategy from latent TB to active TB will also be crucial for improving the quality of life for the entire society.

In the studies finally selected for this review, additional affecting factors to HRQOL of TB, as well as TB treatment outcomes were reported. Quitting smoking, in the Awaisu et al. (2012) study and social support, in the Zarova et al. (2018) study could further increase utility weight. In Kittikraisak et al. (2012), income levels and age groups (under 40 years of age, above) had a significant impact on HRQOL. The subjects of our review that were extracted from finally selected articles are younger patients aged 30–40 compared to general TB patients in South Korea, and the quality of life of TB patients in South Korea may be assessed lower considering the higher proportion of elderly patients in Korea.

The limitations of this review are as follows: First, the heterogeneity among studies tends to be high and the medical system could be different between study countries. Furthermore, the number of studies included in the meta-analysis for the EQ-5D was small, because the selected studies had different value sets or EQ-5D instrument versions in determining utility score. Thus, caution should be taken in interpreting the results of the meta-analysis, also taking into account the characteristics of individual studies and differences between researchers and subjects. However, we presented complementary results using EQ VAS, thus supporting the robustness of the main results. Second, all of the studies had no blinding process as observational studies and could not be compared with controls, so the level of evidence could be weak compared to clinical trials. However, the comparison of HRQOL before and after TB treatment is thought to be valid by using the self-controlled study design. Third, the value set of the EQ-5D is different country by country and utility values are generally affected by the socio-economic factors of the target subjects (Szende et al., 2014), and therefore, this should be taken into account when generalizing the results of this study to Korean patients.

In conclusion, the results of this systematic review study confirmed that the HRQOL of TB patients based on EU-5D utility score was significantly lower compared to that of the general population and successful treatment had a significant effect on the improvement of HRQOL. The HRQOL research on MDR-TB is still insufficient, so further research is needed in the future.

## Data Availability

The original contributions presented in the study are included in the article/Supplementary Material, further inquiries can be directed to the corresponding author.

## Footnotes

1
https://euroqol.org/publications/search-for-eq-5d-in-pubmed/.

## Supplementary Material

Supplementary Table S1. Search terms and results.
