# Supplementary material for: Health-Related Quality of Life Based on EQ-5D Utility Score in Patients With Tuberculosis: A Systematic Review
Source: Front Pharmacol. 2021 Apr 14;12:659675. doi: 10.3389/fphar.2021.659675 (PMC8080025; doi:10.3389/fphar.2021.659675)
Supplement: Supplementary file 1 [file table1.pdf]

Supplementary Material

Supplementary Table S1. Search terms and results

Date : 10 January 2020

Database: PubMed

No	Search terms	Results
#1	"tuberculosis"[Mesh]	189,352
#2	"utility value"[tw] OR "utility values"[tw] OR "utility weight"[tw] OR "utility weights"[tw]	1,694
#3	"preference"[tw] OR "preferences"[tw]	160,839
#4	"quality of life"[tw] OR "quality adjusted life years"[tw] OR QALY[tw] OR "quality of well being"[tw]	327,740
#5	"visual analogue scale"[tw] OR "standard gamble"[tw] OR "time trade off"[tw] or"EQ-5D"	30846
#6	(#2 OR #3 OR #4 OR #5)	501692
#7	(#1 AND #6)	640
#8	#7 Filters: Humans; English	510
#9	#8 Filters: Publication date from 2000/01/01	467

Date: 10 January 2020

Database: EMBASE

No	Search terms	Results
#1	'tuberculosis'/exp OR tuberculosis	338675
#2	'quality of life'	551762
#3	'EQ-5D'	14,629
#4	PREFEREN*	349400
#5	'standard gamble'	1098
#6	'time trade off'	5910
#7	#2 OR #3 OR #4 OR #5 OR #6	895,810
#8	#1 AND #7	3759
#9	#8 AND [article]/lim AND [humans]/lim AND [english]/lim AND [clinical study]/lim AND [2000-2020]/py	972

Date: 10 January 2020

Database: Cochrane

No	Search terms	Results
#1	Tuberculosis	7097
#2	Tuberculosis:ti,ab,kw	0
#3	#1 or #2	7097
#4	"utility value" or "utility values" or "utility weight" or "utility weights"	390
#5	"utility"	162948

#6	"preference" or "preferences"	17373
#7	"quality of life" or "quality adjusted life years" or QALY or "quality of well being"	105071
#8	"visual analogue scale" or "standard gamble" or "time trade off" or "discrete choice experiment"	41353
#9	(#4 or #5 or #6 or #7 or #8)	162948
#10	(#9 and #3), with Publication Year from 2000 to present , with Cochrane Library publication date from Jan 2000 to present, in Trial	369

Date: 17 Febuary 2020

Database: KoreaMed

No	Search terms	Results
#1	Tuberculosis	6426
#2	"utility“	6346
#3	((quality of life) OR quality adjusted life years) OR QALY	4539
#4	(standard gamble) OR time trade off	28
#5	#2 or #3 or #4	10694
#6	5 and # 1, original article, DP:2000:2019	102

Date: 11 Febuary 2020

Database: RISS

No	Search terms (Search terms were applied in Korean)	Results
#1	Tubercuolsis	18055
#2	#1 and (quality of life)	39
#3	#1 and (utility)	30

Date : 1 March 2021

Database: PubMed

No	Search terms	Results
#1	"tuberculosis"[Mesh]	194419
#2	“EQ-5D” OR “EQ5D” OR “EuroQol”	12212
#3	(#1 AND #2)	12
#4	#3 Filters: Humans; English	12
#5	#4 Filters: Publication date from 2000/01/01-2020/1/10	11
